# Editorial: Cell and molecular signaling, and transport pathways involved in growth factor control of synaptic development and function

**DOI:** 10.3389/fnsyn.2015.00008

**Published:** 2015-06-04

**Authors:** Akira Yoshii, Martha Constantine-Paton, Nancy Y. Ip

**Affiliations:** ^1^Department of Anatomy and Cell Biology, and Pediatrics, University of Illinois at ChicagoChicago, IL, USA; ^2^Department of Brain and Cognitive Science, McGovern Institute for Brain Research, Massachusetts Institute of TechnologyCambridge, MA, USA; ^3^McGovern Institute for Brain Research and Massachusetts Institute of TechnologyCambridge, MA, USA; ^4^Department of Biology, Massachusetts Institute of TechnologyCambridge, MA, USA; ^5^Division of Life Science, Hong Kong University of Science and TechnologyHong Kong, China; ^6^Molecular Neuroscience Center and Hong Kong University of Science and TechnologyHong Kong, China; ^7^State Key Laboratory of Molecular Neuroscience, Hong Kong University of Science and TechnologyHong Kong, China

**Keywords:** BDNF, neurotrophic factors, synapse formation, FGF7, FGF22, UNC-5, UNC-6 Netrin

Since the discovery of nerve growth factor (NGF) more than a half century ago (Levi-Montalcini and Cohen, 1960), the prototypic neurotrophin family has included brain derived neurotrophic factor (BDNF), neurotrophin-3 (NT-3), and neurotrophin-4 (NT-4). Neurotrophins bind to the Trk family of receptors, as well as the p75 receptor, to activate multiple intracellular signaling cascades (reviewed by Reichardt, 2006). BDNF receptor tropomyosin receptor kinase B (TrkB) signaling has been extensively studied for its roles in the central nervous system (CNS) ranging from cell survival, axonal and dendritic growth and synapse formation. The pathway mediates long-lasting activity-modulated synaptic changes on excitatory and inhibitory neurons and plays critical roles in circuit development and maintenance. In addition to BDNF, many studies have identified other “growth” or signaling factors in the CNS that play important roles in the development, maintenance, and control of synaptic and circuit function. However, details of the intracellular signaling systems downstream of these events are frequently unexplored. In this Research Topic, we have collected original studies and review articles that present cellular and molecular mechanisms concerning activity-dependent synapse formation and their implications for behavior and brain disorders.

Vadodaria and Jessberger discuss synapse maturation in adult-born dentate granule cells and the role of BDNF-TrkB and several other signaling pathways that activate Cdc42, Rac1, and RhoA (Vadodaria and Jessberger, 2013). These small Rho GTPases regulate polymerization of actin and microtubules, and are consequently involved in aspects of neuronal maturation ranging from cell migration, to dendritic arborization, spine maturation, and synaptic integration of these newborn hippocampal neurons.

Kellner et al. show that BDNF is critical for activity-dependent maintenance of mature spines through F-actin polymerization (Kellner et al., 2014). Integrity of this spine cytoskeleton is also critical for the vesicular transport, carried out by molecular motor proteins. For example, Myosin Va is a plus end actin, vesicular motor protein that carries postsynaptic density protein 95 (PSD-95), Synapse-associated protein 90/postsynaptic density-95-associated protein (SAPAP) and Shank, an essential glutamate receptor scaffold complex along actin to the postsynaptic membrane at the tip of dendritic spines (Hammer and Wagner, 2013; Yoshii et al., 2013). Furthermore, BDNF-TrkB signaling triggers transport of the vesicles containing PSD-95 and its associated complex to postsynaptic membranes via activation of PI3-kinase-Akt pathway (Yoshii and Constantine-Paton, 2007).

BDNF-TrkB signaling pathway also regulate the molecular assembly of synaptic membrane. Zonta and Miniciello discuss the emerging role of lipid raft, the detergent resistant lipid microdomain enriched with cholesterol and sphingolipid, in synaptic plasticity as a result of neurotrophin signaling (Zonta and Minichiello, 2013). When BDNF binds to the TrkB receptor, the ligand-receptor complex shifts to lipid rafts via activation of tyrosine kinase Fyn (Pereira and Chao, 2007; Suzuki et al., 2007). Lipid raft is enriched in postsynaptic membrane, and facilitates localization of palmitoylated proteins such as PSD-95, which is a critical regulator of synaptic plasticity at excitatory synases. Palmitoylation of PSD-95 in the cell body is also mediated by BDNF-TrkB signaling via activation of phospholipase Cγ (PLCγ) and protein kinase C (PKC). The PKC inhibitors chelerythrine as well as a synthetic *z*eta *i*nhibitory *p*eptide (ZIP) designed to block the brain-specific PKC isoform protein kinase Mζ (PKMζ) were used to demonstrate this effect (Yoshii et al., 2011). However, additional studies in the hippocampus began to raise concerns about the specificity of ZIP (Lee et al., 2013; Volk et al., 2013). A follow-up study by Yoshii and Constantine-Paton in this Topic confirmed that, while both chelerythrine and ZIP could suppress the postsynaptic localization of PSD-95, PKMζ knock-down with RNA interference did not exhibit this effect. The result indicates that the ZIP peptide, widely used as a “specific” PKMζ antagonist, may block another PKC variant that is the kinase actually involved in PSD-95 palmitoylation in cell body.

BDNF is critical for mechanisms underlying various modalities of sensory processing, cognition and behaviors. These roles have been studied in hippocampal learning and memory (reviewed by Minichiello, 2009), in maturation and plasticity of the CNS and also in neurological disease (reviewed by Yoshii and Constantine-Paton, 2010). It is not surprising that BDNF is associated with brain disorders such as epilepsy, autism, depression, and schizophrenia since all of these have chronic effects on synaptic function. However, Andersen and Sonntag have studied the effect of juvenile exposure to psychostimulants on the risk of cocaine addiction in adulthood. They found that treatment with methylphenidate, frequently used to improve attention in children, has a long-lasting suppressive effect on cocaine-induced increases in BNDF expression (Andersen and Sonntag, 2014). Further studies will hopefully identify the mechanisms underlying the critical period effect on addiction as well as long term effect of stimulants.

Neurotrophic factors also affect feeding. Maekawa et al. have shown that low BDNF expression in the ventromedial hypothalamus correlates with blood glucose level, increased leptin secretion and eating, and visceral fat accumulation in a type 2 diabetic rat line (Maekawa et al., 2013). The results indicate BDNF and leptin play major roles in central regulation of energy metabolism and dysregulation of the neurotrophin signaling result in obesity.

Harvey discusses that leptin regulates synaptic functions (Harvey, 2013). This dietary hormone activates extracellular-signal-regulated kinase (ERK) and facilitates GluN2B-mediated synaptic depression during early postnatal development while it regulates LTP through PI3-kinase pathway in adult hippocampus (Moult and Harvey, 2011).

It is now clear that variety of synaptogenic growth factors are wider than previously thought. Two articles review diversity of these factors. Williams and Umemori discuss members of the fibroblast growth factor (FGF) family in the context of synaptic development (Williams and Umemori, 2014). FGFs have been shown to organize presynaptic vesicle clustering. Remarkably, FGF7-null mice exhibit a specific deficit in hippocampal inhibitory synapse formation while FGF22-null mice are deficient in excitatory synapses (Terauchi et al., 2010). Furthermore, FGF7-null animals are prone to develop epilepsy after kindling, while FGF22-null are resistant to this seizure induction.

Poon et al. provide a comprehensive review of Netrin, Wnt, transforming growth factor-β (TGF-β), tumor necrosis factor-α (TNF-α), all of which were first identified for their roles other than synaptogenesis (Poon et al., 2013). For example, UNC-6/netrin and its receptor UNC-5 were originally described as axonal guidance molecules. However, UNC-6 and UNC-5 facilitate localization of presynaptic proteins to axons by excluding them from dendrites (Poon et al., 2008). Consequently, these pathway are all involved in regulating axonodendritic polarity.

The field of neuronal growth factors is continuing to grow, and new discoveries, some which are highlighted in this volume, will prompt new questions. For example, do these growth factors work together, competitively or separately? Is there cell-type specificity for each factor? Do they play a deterministic or a modulatory role in synaptic specificity during development ? Advances in genomics and proteomics will help us understand not only single cascade but also multiple signaling pathways as network. Various genetic tools allow spacial and temporal controls of gene expression and neuronal activities (Boyden et al., 2005; Arenkiel and Ehlers, 2009; Konermann et al., 2013). Super-resolution microscopy enables observations of signaling molecules at synapse and organelles in unprecedented manner. Multi-photon microscopy has been invaluable to study a wide range of structures from individual spines to neuronal circuitry. Applications of these new technologies will create exciting opportunities to tackle the above and other questions.

## Conflict of interest statement

The authors declare that the research was conducted in the absence of any commercial or financial relationships that could be construed as a potential conflict of interest.
